# Spatiotemporal Evolution of SARS-CoV-2 Alpha and Delta Variants during Large Nationwide Outbreak of COVID-19, Vietnam, 2021

**DOI:** 10.3201/eid2905.221787

**Published:** 2023-05

**Authors:** Nguyen Thi Tam, Nguyen To Anh, Trinh Son Tung, Pham Ngoc Thach, Nguyen Thanh Dung, Van Dinh Trang, Le Manh Hung, Trinh Cong Dien, Nghiem My Ngoc, Le Van Duyet, Phan Manh Cuong, Hoang Vu Mai Phuong, Pham Quang Thai, Nguyen Le Nhu Tung, Dinh Nguyen Huy Man, Nguyen Thanh Phong, Vo Minh Quang, Pham Thi Ngoc Thoa, Nguyen Thanh Truong, Tran Nguyen Phuong Thao, Dao Phuong Linh, Ngo Tan Tai, Ho The Bao, Vo Trong Vuong, Huynh Thi Kim Nhung, Phan Nu Dieu Hong, Le Thi Phuoc Hanh, Le Thanh Chung, Nguyen Thi Thanh Nhan, Ton That Thanh, Do Thai Hung, Huynh Kim Mai, Trinh Hoang Long, Nguyen Thu Trang, Nguyen Thi Hong Thuong, Nguyen Thi Thu Hong, Le Nguyen Truc Nhu, Nguyen Thi Han Ny, Cao Thu Thuy, Le Kim Thanh, Lam Anh Nguyet, Le Thi Quynh Mai, Tang Chi Thuong, Le Hong Nga, Tran Tan Thanh, Guy Thwaites, H. Rogier van Doorn, Nguyen Van Vinh Chau, Thomas Kesteman, Le Van Tan, for the OUCRU COVID-19 Research Group

**Affiliations:** Oxford University Clinical Research Unit, Hanoi, Vietnam (N.T. Tam, T.S. Tung, N.T. Trang, N.T.H. Thuong, T. Kesteman, H. R. van Doorn);; National Hospital for Tropical Diseases, Hanoi (P.N. Thach, V.D. Trang, L.V. Duyet, P.M. Cuong);; Vietnam Military Medical University, Hanoi (T.C. Dien);; National Institute of Hygiene and Epidemiology, Hanoi (H.V.M. Phuong, L.T.Q. Mai, P.Q. Thai);; Hue National Hospital, Hue, Vietnam (P.N.D. Hong, L.T.P. Hanh);; Da Nang Center for Disease Control, Da Nang, Vietnam (L.T. Chung, N.T.T. Nhan, T.T. Thanh);; Pasteur Institute, Nha Trang, Khanh Hoa, Vietnam (D.T. Hung, H.K. Mai, T.H. Long);; Oxford University Clinical Research Unit, Ho Chi Minh City, Vietnam (N.T. Anh, N.T.T. Hong, L.N.T. Nhu, N.T.H. Ny, T.T. Thanh, C.T. Thuy, L.K. Thanh, L.A. Nguyet, G. Thwaites, L.V. Tan);; Hospital for Tropical Diseases, Ho Chi Minh City (N.T. Dung, L.M. Hung, N.L.N. Tung, D.N.H. Man, N.M. Ngoc, N.T. Phong, V.M. Quang, P.T.N. Thoa, N.T. Truong, T.N.P. Thao, D.P. Linh, N.T. Tam, H.T. Bao, V.T. Vuong, H.T.K. Nhung);; Department of Health, Ho Chi Minh City, (N.V.V. Chau, T.C. Thuong);; Ho Chi Minh City Center for Disease Control, Ho Chi Minh City (L.H. Nga);; University of Oxford, Oxford, UK (H.R. van Doorn, G. Thwaites, L.V. Tan)

**Keywords:** COVID-19, coronavirus disease, SARS-CoV-2, severe acute respiratory syndrome coronavirus-2, coronaviruses, viruses, coronavirus disease, respiratory infections, Alpha variant, Delta variant, spatiotemporal evolution, outbreak, zoonoses, Vietnam

## Abstract

We analyzed 1,303 SARS-CoV-2 whole-genome sequences from Vietnam, and found the Alpha and Delta variants were responsible for a large nationwide outbreak of COVID-19 in 2021. The Delta variant was confined to the AY.57 lineage and caused >1.7 million infections and >32,000 deaths. Viral transmission was strongly affected by nonpharmaceutical interventions.

After successfully controlling SARS-CoV-2 transmission in 2020 (*1*), Vietnam experienced a large nationwide outbreak of infection with SARS-CoV-2 in 2021. This outbreak was characterized by 2 distinct phases: January–April, with 1,632 infections and no deaths, and May–December, with 1,727,398 infections and 32,359 deaths (*2*).

Genomic surveillance has been one of the top priorities of the World Health Organization and has generated major insights into the spatiotemporal evolution of SARS-CoV-2 (*3*), which are critical for pandemic response. However, most of the studies published about genetic evolution of SARS-CoV-2 are based on datasets from high-income countries with relatively open borders (*4**–**6*), and little is known about the transmission dynamics of SARS-CoV-2 in countries such as Vietnam where strict nonpharmaceutical interventions were implemented. We analyzed the spatiotemporal evolution of SARS-CoV-2 in Vietnam during 2021 and mapped patterns of viral evolution and diffusion against the public health measures implemented during the study period.

## The Study

The study was conducted at the National Hospital for Tropical Diseases in Hanoi, Vietnam, and the Hospital for Tropical Diseases in Ho Chi Minh City, Vietnam. Both are tertiary referral hospitals for COVID-19 patients in northern (hospital in Hanoi) and southern (hospital in Ho Chi Minh City) Vietnam. The laboratories of the 2 hospitals were responsible for SARS-CoV-2 diagnosis and sequencing in Vietnam. We compiled detailed study methods, including public health measures (Appendix Figure 1), and demographic features of study participants (Table).

**Table:** Demographics of study participants for spatiotemporal evolution of SARS-CoV-2 Alpha and Delta variants during large nationwide outbreak of COVID-19, Vietnam, 2021*

Characteristic	No. sequences, n = 1,303	Alpha, n = 74	AY.57, n = 1,212
Age, y			
Median (Q1‒Q3)	43 (29‒61)	35 (30–55)	44 (29‒61)
Missing	38 (2.9)	31 (41.9)	2 (0.2)
Sex			
M	689 (52.9)	22 (29.7)	661 (54.5)
F	578 (44.4)	21 (28.4)	551 (45.5)
Unknown	36 (2.8)	31 (41.9)	
Region			
Central	120 (9.2)	24 (32.4)	94 (7.8)
Northern	504 (38.7)	44 (59.5)	450 (37.1)
Southern	679 (52.1)	6 (8.1)	668 (55.1)

*Values are no. (%) unless indicated otherwise. Characteristics of A.23.1 variant‒infected cases were previously reported (*7*). Q, quartile.

We selected 1,365 nasopharyngeal throat swab specimens for whole-genome sequencing and obtained 1,303 complete genome sequences. We detected no recombinants. Most obtained sequences belonged to Delta variant (93.8%, n = 1,222), followed by Alpha (5.7%, n = 74), A.23.1 (0.4%, n = 5), and B.1.637 (0.2%, n = 2) variants. Of the Delta sequences, 1,212 (99.2%) were assigned to AY.57 lineage by PANGO lineage (*8*). The remaining were assigned to AY.23 and AY.79 (n = 3 each), AY.6, AY.38, AY.85, and B.1.617.2 (n = 1 each).

We temporally documented the Alpha and A.23.1 sequences during January‒May 2021. We detected the first 3 Delta sequences, including 2 AY.57 and 1 B.1.617.2, in late April 2021. From June on, Delta was the only variant detected, coinciding with an upsurge in the number of infections and deaths during the 2021 outbreak (Appendix Figure 2).

**Figure 1:**
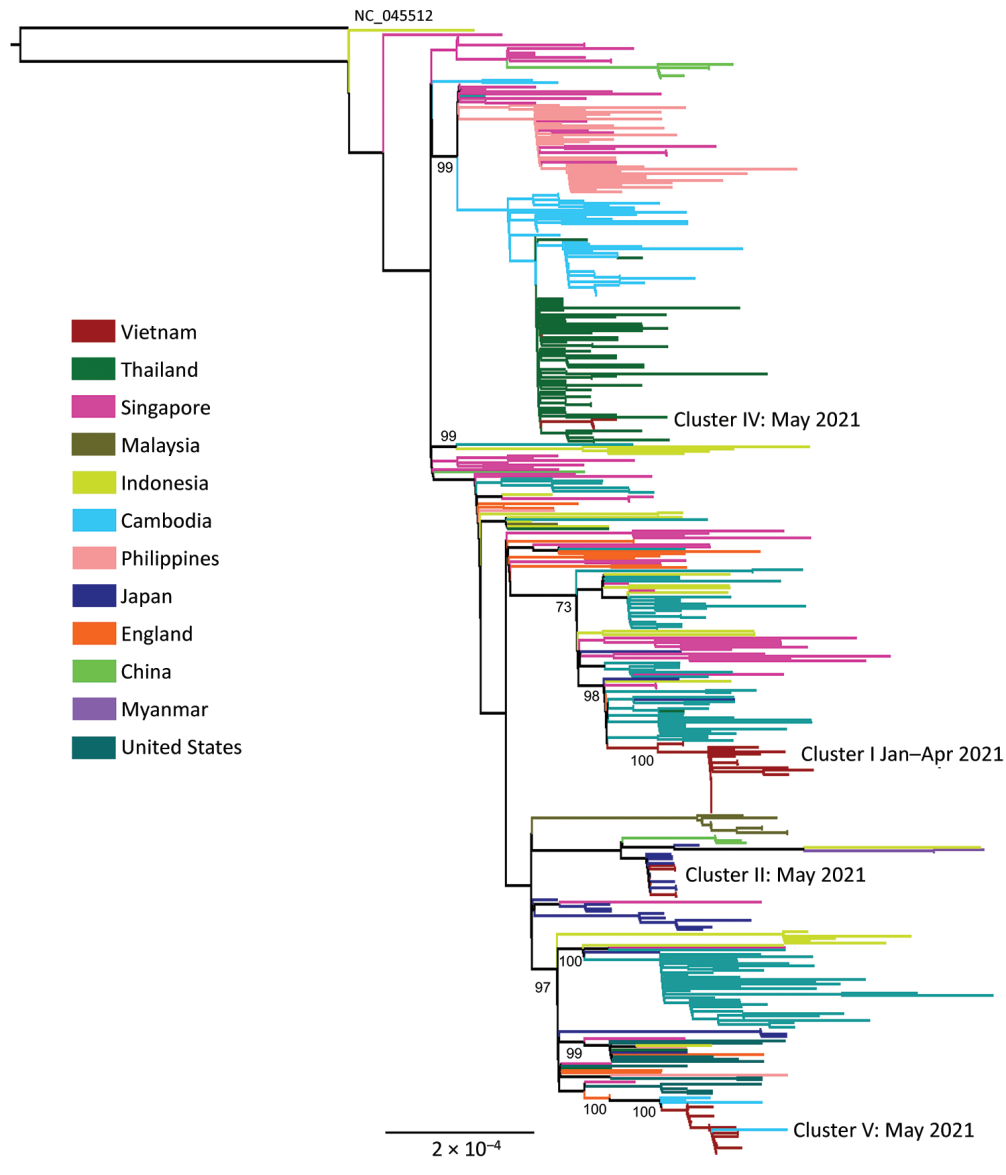
Maximum-likelihood tree of SARS-CoV-2 Alpha and Delta variants during large nationwide outbreak of COVID-19, Vietnam, 2021. Complete coding sequences of Alpha variant viruses circulating in Vietnam in 2021 are shown. Phylogenetic clusters were named accordingly to community clusters recorded during the study period shown in Appendix Figure 1). Numbers along branches are bootstrap values. Cluster II was linked with a traveler from Japan (Appendix Figure 1). Outgroup was the SARS-CoV-2 wild-type strain (GenBank accession no. NC_0455122).

Maximum-likelihood phylogenetic analysis of Alpha variant sequences showed that they were closely related to the contemporary sequences detected in the region and clustered into 4 major groups, corresponding to the sporadic community transmission clusters detected in northern, central, and southern Vietnam in early 2021 (Figure 1). This finding suggested that multiple importations and exportations of the Alpha variant into Vietnam occurred during January‒May 2021.

Because of the dominance of the AY.57 lineage, and the small number of AY.57 sequences reported outside Vietnam, and especially in the nearby region (Cambodia, n = 5; Thailand, n = 5; Laos, n = 0; Singapore, n = 5), we focused our phylogeographic analysis on the 1,212 Delta AY.57/1.303 sequences obtained from Vietnam. After we removed identical, low-quality, and outlier sequences, as suggested by TempEst software, (https://tempest-solutions.com), 748 non-identical sequences were available for analysis.

**Figure 2:**
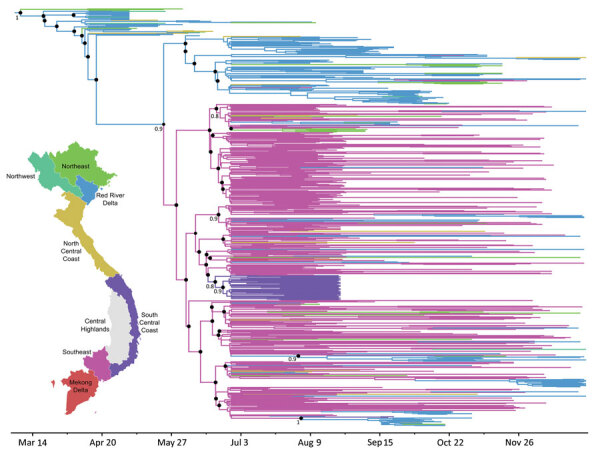
Spatiotemporal evolution of SARS-CoV-2 Alpha and Delta variants during large nationwide outbreak of COVID-19, Vietnam, 2021. Maximum clade credibility tree illustrates the phylogeography of AY.57 lineage in Vietnam in 2021, Branches are color-coded according to locations of sampling shown on map. Numbers along branches are bootstrap values. Posterior probabilities >0.8% and state probabilities >75% (black circles) are indicated at all nodes.

Results confirmed that AY.57 viruses were introduced into the northeastern region in early 2021 (Figure 2) probably by a single introduction event (Appendix Figure 4). The estimated time to the most recent common ancestor was March 14, 2021 (95% CI February 22, 2021‒April 8, 2021), shortly after the discovery of the Delta variant in November 2020. In the following months, the northeastern and Red River delta regions then acted as a source seeding the virus to neighboring and southeastern provinces, with limited viral dispersal between provinces/cities (Figure 2). During July‒December 2021, the southeastern region was the main source, seeding the virus back to the northern region and the rest of Vietnam. In addition, we observed the establishment of multiple localized clusters of AY.57 lineage elsewhere in the southcentral coastal region and the Red River delta (Figure 2).

**Figure 3:**
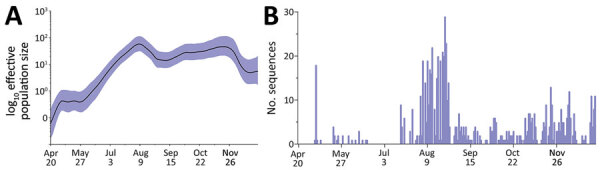
Genetic diversity and distribution of SARS-CoV-2 AY.57 lineage during large nationwide outbreak of COVID-19, Vietnam, 2021. A) Bayesian skyride plot illustrating the relative genetic diversity of AY.57 lineage in Vietnam during 2021. Purple shading indicates 95% highest posterior density interval. B) Distribution of the number of AY.57 sequences used for analysis over the study period.

A Bayesian Skyride showed a sharp increase in genetic diversity during April‒August 2021 (Figure 3, panel A), reflecting the expansion of the AY.57 lineage across the country, paralleling the start of the large nationwide outbreak from May onward (Appendix Figures 1, 2). In the following months, the viral population size remained relative stable, despite some fluctuations in the number of viral sequences obtained (Figure 3, panel B), followed by a slight decrease in the genetic diversity during November. The estimated mean of the nonsynonymous to synonymous substitution (dN/dS) value was 0.86, albeit it varied across the genomes (Appendix Table 1), and the evolutionary rate of AY.57 coding sequences was 5.29 × 10^−4^ substitutions/site/year (95% CI 4.966 × 10^−4^ to 5.639 × 10^−4^ substitutions/site/year).

## Conclusions

We showed that the Alpha and especially Delta variants were the main causes of SARS-CoV-2 outbreaks of >1.7 million infections and >32,000 deaths in Vietnam during 2021. The Alpha variant was introduced into Vietnam during early 2021 by different importation events but only caused sporadic community outbreaks until May, when a second wave associated with the Delta variant predominated from June on. The viruses of the Delta variant were confined to AY.57 lineage and were responsible for the major wave in 2021, probably by a single introduction event. Viral movement between provinces was not apparent. During the study period, nearly 200 sublineages of the Delta variant were documented worldwide (*9*), and in other countries, such as the United Kingdom and the United States, where use of nonpharmaceutical measures was relaxed, viral dispersal across the localities was more apparent (*10**,**11*). The rigorous containment approach applied in Vietnam in 2021, with limited domestic travels and tight border controls, was probably the key factor determining the localization of a single AY.57 lineage in Vietnam and its limited dispersal across the country.

The sharp increase in the relative genetic diversity of the AY.57 lineage during April‒July 2021 marked the start of the nationwide outbreak in subsequent months despite in-country lockdown measures. Although nonpharmaceutical interventions were sufficient to prevent uncontrolled community transmission in 2020 (*1*), they were not sufficient after introduction of the Delta variant. This finding was probably caused by the much higher transmissibility of the Delta variant and the immunologically naive population in Vietnam at the time. During April‒July 2021, only <1% of 97 million persons in Vietnam were vaccinated against SARS-CoV-2 (*12*).

Although our estimated evolutionary rate was AY.57-lineage specific, the result was within the range of previously estimated values for the Delta variant more broadly (*10*; N. Benazi, Institut Pasteur of Algeria, and S. Bounab, University of M’sila, pers. comm., email, 2023 Mar 1). This finding points to a fast evolution of the AY.57 lineage in Vietnam during the study. Although the role of population immune landscapes in shaping the evolution of SARS-CoV-2 merits further research, a recent report showed that vaccines had played a role in selective adaptation of the SARS-CoV-2 Delta variant (*13*).

The potential bias toward our referral-hospital based sampling approach represents a limitation of our study, which might have failed to comprehensively capture the genetic diversity of the pathogen. However, this limitation was probably modest given that our results are consistent with sequences uploaded to GISAID (https://www.gisaid.org) (*9*).

In conclusion, we report how rigorous public health measures in Vietnam influenced the introduction and spread of the Alpha and Delta variants during the large nationwide outbreak of COVID-19 in 2021. Genomic surveillance is critical to inform pandemic response.

Appendix Additional information on spatiotemporal evolution of SARS-CoV-2 Alpha and Delta variants during large nationwide outbreak of COVID-19, Vietnam, 2021.
