# Supplementary material for: Spatiotemporal Evolution of SARS-CoV-2 Alpha and Delta Variants during Large Nationwide Outbreak of COVID-19, Vietnam, 2021
Source: Emerg Infect Dis. 2023 May;29(5):1002–6. doi: 10.3201/eid2905.221787 (PMC10124647; doi:10.3201/eid2905.221787)
Supplement: Appendix — Additional information on spatiotemporal evolution of SARS-CoV-2 Alpha and Delta variants during large nationwide outbreak of COVID-19, Vietnam, 2021. [file 22-1787-Techapp-s1.pdf]

Article DOI: https://doi.org/10.3201/eid2905.221787

EID cannot ensure accessibility for Supplemental Materials supplied by authors. Readers who have difficulty accessing supplementary content should contact the authors for assistance.

Spatiotemporal Evolution of SARS-CoV-2 Alpha and Delta Variants during Large Nationwide Outbreak of COVID-19, Vietnam, 2021

Appendix

Vietnam COVID-19 Containment Approach in 2021

After successfully controlling community transmission during the first 8 months in 2020, Vietnam cautiously reopened its borders, allowing some repatriation flights, while steadily lifting in-country travel restrictions (*1*). However, following the emergence of new SARS-CoV-2 variants Alpha and Delta variants in late, Vietnam suspended all inbound flights from countries reporting the detection of community transmission associated with these two variants. Additionally, all travelers entering Vietnam were subjected to 14-day isolation, and testing on the day of arrival and on day 14 of quarantine. Yet, after 28 days of no community transmission, on 18th January 2021, two clusters of SARS-CoV-2 infection with epidemiologic links were detected in two neighboring provinces in the north of Vietnam, Hai Duong and Quang Ninh (*2*). This was followed by the detection of several other community transmission clusters of unclear origin in several provinces across the country in subsequent months before the start of the 2021 major outbreak (Appendix Figure 1). At the national level, the responses were initially targeted e.g., lockdown at community/province/city level where a community transmission cluster was detected. However, in response to the escalation of the outbreak starting in July 2021 (*3*), Vietnam suspended all arriving international flights in May 2021, and re-applied in-country travel restriction (Appendix Figure 1) until 10th October 2021 when nearly 70% of the eligible population had received at least 1 dose of vaccine (*3*). In parallel, under the zero COVID-19 policy, Vietnam implemented the meticulous contact tracing and mass testing of the cases and their contacts conducted by provincial Centers for Diseases Control (CDC). This had enabled

accurate identification of cases of community transmission origin alongside the demographic data for analysis (*4*).

Nasopharyngeal Swabs and Sample Selection for Sequencing

The laboratories of National Hospital for Tropical Diseases (NHTD) in Hanoi, and the Hospital for Tropical Diseases (HTD) in Ho Chi Minh City, Vietnam were responsible for SARS-CoV-2 diagnosis and sequencing in Vietnam. Therefore, nasopharyngeal swab (NPS) samples submitted to NHTD and HTD laboratories for testing and sequencing were either from provincial CDC or from inpatients being treated at these two hospitals.

To increase the chance of successfully obtaining the virus genome, we first applied a preselection criterion based on the cycle threshold (Ct) value of the tested samples generated by the Lightmix Modular SARS-CoV-2 RdRp/E gene assay (Tib Molbiol, Berlin, Germany) (*5*). This assay could detect both Alpha and Delta variants without compromising the sensitivity (*6*). Accordingly, at NHTD, only NPS samples with a cycle threshold (Ct) value ≤30 for the RdRp gene were eligible, while at HTD, a sample with Ct value of ≤25 for the E gene was included. Additionally, because of the availability of the resources, between January and June 2021 when community transmission remained limited, our approach focused on new community clusters detected through contact tracing under zero-COVID strategy. Between July and December 2021 during which community transmission was escalating, the selection of samples for sequencing was carried out by using WHO recommendations (*7*). Epidemiologic data, and data on infections and deaths were retrieved from e-hospital record or provided by the National Institute of Hygiene and Epidemiology and the Vietnamese Ministry of Health. Here, we focused our analysis on sequences obtained cases of locally acquired infection, and generated by NHTD or HTD laboratories, of which we had accurate sampling date and demographic data.

Our study formed part of the national COVID-19 response and was approved by the local Institutional Review Board (approval no. 2221/QĐ-BVBNĐ at the Hospital for Tropical Diseases in Ho Chi Minh City and no. 17–2022/HĐĐĐ -NĐTW at the National Hospital for Tropical Diseases in Hanoi) and Oxford Tropical Research Ethics Committee (approval no. 557–21). Since only deidentified nasopharyngeal swabs (NPS) were used, the need for individual informed consent was waived.

RNA Extraction, Whole-Genome Sequencing, and Sequence Assembly

Total RNA was extracted from NPS by using QIAamp viral RNA mini kit (Qiagen, Hilden, Germany) following the manufacturer's instructions, and finally eluted in 50µl of elution buffer (provided with the extraction kit). Whole genome amplification was performed on the extracted RNAby using either the long pooled amplicons protocol, developed by the University of Sydney (*8,9*), or the ARTIC V3 protocol (*10*) on an Illumina MiSeq platform as previously described. Library preparation was carried out by using the Nextera XT Library preparation kit (Illumina, USA), followed by library quantification by using KAPA Library Quant Kit (Kapa Biosystems, Wilmington, MA, USA), according to the manufacturer's instructions. The prepared library was sequenced by using iSeq reagent kit V2 (300 cycles) on a Miseq platform (Illumina). For each run, tested samples were multiplexed and differentiated by double indexes by using IDT-ILMN Nextera DNA UD indexes (IDT).

Sequence assembly of the obtained sequencing data was carried out by using a reference-based mapping approach available in CLC genomics workbench (v.21.0.4) and Geneious 8.1.5 (Biomatters, San Francisco, CA, USA). This method involved mapping of sequencing output of individual samples to a reference genome (WuHan-Hu-1: NC_045512, Alpha: EPI_ISl_905782, Delta: EPI_ISL_1942165), followed by manual editing of the obtained consensus to ensure the accuracy of the results, as described previously (*8*). The consensus sequences generated in this study were submitted to the National Center for Biotechnology Information under the assigned accession numbers ON458864-ON459533, ON459545-ON459608, ON755375-ON755859, OQ415286- OQ415315, and OP647358-OP647411, and GISAID with assigned numbers: EPI_ISL_10079201-EPI_ISL_10079251, EPI_ISL_2455221-EPI_ISL_2458062, EPI_ISL_4503976-EPI_ISL_4504960, EPI_ISL_4748289-EPI_ISL_4748342, EPI_ISL_4942690-EPI_ISL_4942691, EPI_ISL_4969141-EPI_ISL_4969175, EPI_ISL_5098167-EPI_ISL_5098169, EPI_ISL_5458630-EPI_ISL_5458977, EPI_ISL_6773765-EPI_ISL_6773813, EPI_ISL_7368716-EPI_ISL_7368772, EPI_ISL_7648835-EPI_ISL_7648869, EPI_ISL_7650414-EPI_ISL_7650455, EPI_ISL_5945255- EPI_ISL_5945295, EPI_ISL_6388348- EPI_ISL_6388497, EPI_ISL_ 7195776- EPI_ISL_7196062, EPI_ISL_7204787- EPI_ISL_7204791, EPI_ISL_1273214, EPI_ISL_16828666 –EPI_ISL_16828699, EPI_ISL_17016415-EPI_ISL_17016441,

EPI_ISL_17027400-EPI_ISL_17027401, EPI_ISL_17016442-EPI_ISL_17016449, EPI_ISL_4503984.

Classifications of SARS-CoV-2 Variants

SARS-CoV-2 variant classification of the obtained consensuses was determined by using PANGO lineage (*11*) with pangolin v4.1.2 and pangolin-data v1.13 (*12*). The analysis was carried out by using the Ultrafast Sample Placement on Existing Trees option to assign the lineage based on the nearest lineage on existing global tree. Sequence alignment was carried out by using the tool available on Nextclade (*13*) and Minimap2 (*14*). Recombination detection was inferred by using a combination of Freyja v 1.3.10 (*15*) and sc2rf (*16*).

Maximum-Likelihood Framework to Study Genetic Relatedness of Alpha Variant Sequences

To explore the phylogenetic relationship of Vietnamese Alpha variant sequences obtained as part of the present study, we used a dataset consisting of the complete coding region (29,408 bp) of the obtained sequences and Alpha variant sequences randomly selected from those produced from the region and beyond (The U.S. and the UK) during the study period submitted GISIAD. We applied maximum likelihood (ML) method by using the TIM2 nt substitution model with invariant for Alpha variant as suggested by IQ tree (*17*). Support for individual nodes was assessed by using a bootstrap procedure with 1,000 replicates. To assess the placement of the Vietnamese variants in the context of global sequences, we used the phylogenetic framework incorporated in NextClade by using default setting (*13*), i.e., taking into account representatives of global sequences submitted to GISAID (Date of accession: 27 October 2022). Phylogenetic clusters were manually inspected by naked eyes.

Maximum-Likelihood Phylogenetics, Phylogeography, and Phylodynamics Analysis of Delta Variant Sequences

In addition to applying PANGO lineage tool to classify the viral lineages of the Delta variant sequences as detailed above, we used maximum likelihood method and NextClade based phylogenetic analysis frameworks to assess the relatedness of the Delta sequences at national and global scale, respectively. For the former, we applied UNREST+F0+R4 as suggested by IQ tree (*17*). Support for individual nodes was assessed by using a bootstrap procedure with 1,000 replicates. For the latter, we used default setting as outline above.

To study the phylogeographics and phylodynamics of AY.57 lineage, the main lineage detected in Vientam in 2021, we applied Bayesian phylogenetic interference in BEAST v1.10.4 (*18*). We first excluded identical sequences and sequences of low quality (e.g., internal gaps). We then used TempEst 1.5 to assess the temporal signal of the dataset (*19*). Subsequently, we excluded the sequences not conforming to a linear evolutionary pattern as suggested by TempEst. For phylogeographic analysis, we divided Vietnam into eight major geographic regions according to key economic zones (Northeast, Northwest, Red River Delta, North Central Coast, South Central Coast, Highland, Southeast and Mekong Delta) (*20*). Small sample sizes from individual provinces precluded phylogeographic analyses at a finer spatial scale. We used a Bayesian Markov chain Monte Carlo framework (available in BEAST) with 1 billion steps and sampling every 100,000 steps by using the general time reversible (GTR) nucleotide substitution model with invariant (as suggested by IQ-TREE to be the best-fit model) under an uncorrelated relaxed clock model (*21*), and a Bayesian skygride coalescent tree prior (10 groups) (*22*). We assessed convergence by using Tracer version 1.5 (*23*). We selected a burn-in threshold of 10% and accepted effective sample size values above 200. Maximum-clade credibility (MCC) tree was then summarized with TreeAnnotator (available in the BEAST package) and visualized in Figtree version 1.4.2 (*24*).

To assess the effective population size trajectory, we applied Bayesian Skyride model by using the above framework. Finally, we used codon-based method (HyPhy) available in MEGA5 to measure the selection pressure on coding sequences of the pathogen genome, by estimating the ratio of nonsynonymous to synonymous substitution (dN/dS) at gene-wide level (*25*). The Hyphy used the joint maximum-likelihood reconstructions of ancestral states under a Muse-Gaut model of codon substitution.

References

1. Van Tan L. COVID-19 control in Vietnam. Nat Immunol. 2021;22:261. PubMed https://doi.org/10.1038/s41590-021-00882-9

2. Chau NVV, Hong NTT, Ngoc NM, Anh NT, Trieu HT, Nhu LNT, et al.; for OUCRU COVID-19 research group. Rapid whole-genome sequencing to inform COVID-19 outbreak response in Vietnam. J Infect. 2021;82:276–316. PubMed https://doi.org/10.1016/j.jinf.2021.03.017

3. WHO. COVID-19 in Viet Nam Situation Report 101, accessed on 24 October 2022. available at https://www.who.int/vietnam/internal-publications-detail/covid-19-in-viet-nam-situation-report-101, 2022.

4. Van Vinh Chau N, Lam VT, Dung NT, Yen LM, Minh NNQ, Hung LM, et al.; Oxford University Clinical Research Unit COVID-19 Research Group. The Natural History and Transmission Potential of Asymptomatic Severe Acute Respiratory Syndrome Coronavirus 2 Infection. Clin Infect Dis. 2020;71:2679–87. PubMed https://doi.org/10.1093/cid/ciaa711

5. Corman VM, Landt O, Kaiser M, Molenkamp R, Meijer A, Chu DK, et al. Detection of 2019 novel coronavirus (2019-nCoV) by real-time RT-PCR. Euro Surveill. 2020;25:2000045. PubMed https://doi.org/10.2807/1560-7917.ES.2020.25.3.2000045

6. Mögling R, Fischer C, Stanoeva KR, Melidou A, Almeida Campos AC, Drosten C, et al. Sensitivity of Detection and Variant Typing of SARS-CoV-2 in European Laboratories. J Clin Microbiol. 2022;60:e0126122. PubMed https://doi.org/10.1128/jcm.01261-22

7. Organization WH. Operational considerations to expedite genomic sequencing component of GISRS surveillance of SARS-CoV-2. WHO. 1–10. 2021.

8. Nguyen TT, Pham TN, Van TD, Nguyen TT, Nguyen DTN, Le HNM, et al.; OUCRU COVID-19 Research Group. Genetic diversity of SARS-CoV-2 and clinical, epidemiological characteristics of COVID-19 patients in Hanoi, Vietnam. PLoS One. 2020;15:e0242537. PubMed https://doi.org/10.1371/journal.pone.0242537

9. Eden J-S, Sim E. SARS-CoV-2 Genome Sequencing Using Long Pooled Amplicons on Illumina Platforms. Available on https://www.protocols.io/view/sars-cov-2-genome-sequencing-using-long-pooled-amp-kxygxeob4v8j/v1. 2020.

10. Quick, J., nCoV-2019 sequencing protocol V.3. 2020.

11. Rambaut A, Holmes EC, O'Toole Á, Hill V, McCrone JT, Ruis C, et al. A dynamic nomenclature proposal for SARS-CoV-2 lineages to assist genomic epidemiology. Nat Microbiol. 2020;5:1403–7. PubMed https://doi.org/10.1038/s41564-020-0770-5

12. https://github.com/cov-lineages/pangolin.

13. Aksamentov I, Roemer C, Hodcroft E, Neher R. Nextclade: clade assignment, mutation calling and quality control for viral genomes. J Open Source Softw. 2021;6:3773. https://doi.org/10.21105/joss.03773

14. Li H. Minimap2: pairwise alignment for nucleotide sequences. Bioinformatics. 2018;34:3094–100. PubMed https://doi.org/10.1093/bioinformatics/bty191

15. Freyja v 1.3.10, available at https://github.com/andersen-lab/Freyja.

16. Sc2rf - SARS-Cov-2 Recombinant Finder, available at https://github.com/lenaschimmel/sc2rf.

17. Nguyen LT, Schmidt HA, von Haeseler A, Minh BQ. IQ-TREE: a fast and effective stochastic algorithm for estimating maximum-likelihood phylogenies. Mol Biol Evol. 2015;32:268–74. PubMed https://doi.org/10.1093/molbev/msu300

18. Suchard MA, Lemey P, Baele G, Ayres DL, Drummond AJ, Rambaut A. Bayesian phylogenetic and phylodynamic data integration using BEAST 1.10. Virus Evol. 2018;4:vey016. PubMed https://doi.org/10.1093/ve/vey016

19. Rambaut A, Lam TT, Max Carvalho L, Pybus OG. Exploring the temporal structure of heterochronous sequences using TempEst (formerly Path-O-Gen). Virus Evol. 2016;2:vew007. PubMed https://doi.org/10.1093/ve/vew007

20. https://www.vietnam-briefing.com/news/vietnams-regions-key-economic-zones.html/ Access date: 3rd March 2023.

21. Drummond AJ, Ho SY, Phillips MJ, Rambaut A. Relaxed phylogenetics and dating with confidence. PLoS Biol. 2006;4:e88. PubMed https://doi.org/10.1371/journal.pbio.0040088

22. Minin VN, Bloomquist EW, Suchard MA. Smooth skyride through a rough skyline: Bayesian coalescent-based inference of population dynamics. Mol Biol Evol. 2008;25:1459–71. PubMed https://doi.org/10.1093/molbev/msn090

23. Available at http://tree.bio.ed.ac.uk/software/tracer/.

24. Figtree version 1.4.2, available at (http://tree.bio.ed.ac.uk/software/figtree).

25. Tamura K, Peterson D, Peterson N, Stecher G, Nei M, Kumar S. MEGA5: molecular evolutionary genetics analysis using maximum-likelihood, evolutionary distance, and maximum parsimony methods. Mol Biol Evol. 2011;28:2731–9. PubMed https://doi.org/10.1093/molbev/msr121

Appendix Table 1. Breakdown dN/dS ratios for specific coding regions

Gene	CDS	ORF1a	ORF1b	S	ORF3a	E	M	ORF6	ORF7a	ORF7b	ORF8	N
dN/dS	0.86	0.69	1.45	0.59	1.44	1.14	0.27	0.86	1.3	0.82	0.37	0.53

Appendix Table 2. GISAID numbers and geographic locations of sequences used for analysis in the present study

GISAID number	Location
EPI_ISL_2405168	China
EPI_ISL_2405175	China
EPI_ISL_16405160	China
EPI_ISL_9910213	China
EPI_ISL_9910219	China
EPI_ISL_1121993	China
EPI_ISL_9910211	China
EPI_ISL_2432955	China
EPI_ISL_2432957	China
EPI_ISL_11799982	China
EPI_ISL_2432956	China
EPI_ISL_2405171	China
EPI_ISL_1098602	Cambodia
EPI_ISL_1098604	Cambodia
EPI_ISL_1098606	Cambodia
EPI_ISL_1098608	Cambodia
EPI_ISL_1532810	Cambodia
EPI_ISL_1532811	Cambodia
EPI_ISL_1532815	Cambodia
EPI_ISL_1534538	Cambodia
EPI_ISL_1534543	Cambodia
EPI_ISL_1711976	Cambodia
EPI_ISL_1711977	Cambodia
EPI_ISL_1711983	Cambodia
EPI_ISL_1711997	Cambodia
EPI_ISL_1818952	Cambodia
EPI_ISL_1818965	Cambodia
EPI_ISL_1969674	Cambodia
EPI_ISL_1969689	Cambodia
EPI_ISL_2106235	Cambodia
EPI_ISL_2106238	Cambodia
EPI_ISL_2106241	Cambodia
EPI_ISL_2106249	Cambodia
EPI_ISL_2106272	Cambodia
EPI_ISL_2106275	Cambodia
EPI_ISL_2106280	Cambodia
EPI_ISL_2106282	Cambodia
EPI_ISL_2231565	Cambodia
EPI_ISL_2231569	Cambodia
EPI_ISL_2231572	Cambodia
EPI_ISL_2231574	Cambodia
EPI_ISL_2231576	Cambodia
EPI_ISL_2231583	Cambodia
EPI_ISL_2231585	Cambodia
EPI_ISL_2231588	Cambodia
EPI_ISL_2343208	Cambodia
EPI_ISL_2343220	Cambodia
EPI_ISL_2343225	Cambodia
EPI_ISL_2343230	Cambodia
EPI_ISL_2343236	Cambodia
EPI_ISL_2343241	Cambodia
EPI_ISL_2343242	Cambodia

GISAID number	Location
EPI_ISL_2343249	Cambodia
EPI_ISL_2343251	Cambodia
EPI_ISL_2406462	Cambodia
EPI_ISL_2406465	Cambodia
EPI_ISL_2406469	Cambodia
EPI_ISL_2406479	Cambodia
EPI_ISL_2547286	Cambodia
EPI_ISL_4489759	Cambodia
EPI_ISL_4503196	Cambodia
EPI_ISL_1118931	Indonesia
EPI_ISL_1169048	Indonesia
EPI_ISL_1169049	Indonesia
EPI_ISL_1415427	Indonesia
EPI_ISL_1415898	Indonesia
EPI_ISL_1416191	Indonesia
EPI_ISL_1824606	Indonesia
EPI_ISL_2233088	Indonesia
EPI_ISL_2258213	Indonesia
EPI_ISL_2262257	Indonesia
EPI_ISL_2262261	Indonesia
EPI_ISL_2382408	Indonesia
EPI_ISL_2500465	Indonesia
EPI_ISL_2500467	Indonesia
EPI_ISL_2500468	Indonesia
EPI_ISL_2617521	Indonesia
EPI_ISL_2617531	Indonesia
EPI_ISL_2617533	Indonesia
EPI_ISL_2631491	Indonesia
EPI_ISL_2854698	Indonesia
EPI_ISL_2854714	Indonesia
EPI_ISL_2931788	Indonesia
EPI_ISL_2987647	Indonesia
EPI_ISL_3070877	Indonesia
EPI_ISL_3070893	Indonesia
EPI_ISL_3070899	Indonesia
EPI_ISL_3278297	Indonesia
EPI_ISL_5328539	Indonesia
EPI_ISL_7550108	Indonesia
EPI_ISL_1787318	Malaysia
EPI_ISL_1787319	Malaysia
EPI_ISL_2342546	Malaysia
EPI_ISL_3246353	Malaysia
EPI_ISL_3246393	Malaysia
EPI_ISL_3246395	Malaysia
EPI_ISL_3246401	Malaysia
EPI_ISL_3356277	Malaysia
EPI_ISL_3356278	Malaysia
EPI_ISL_934424	Malaysia
EPI_ISL_2592630	Myanmar
EPI_ISL_2595725	Myanmar
EPI_ISL_953385	Singapore
EPI_ISL_1524793	Singapore
EPI_ISL_1543968	Singapore
EPI_ISL_1524785	Singapore
EPI_ISL_1652099	Singapore
EPI_ISL_1620138	Singapore
EPI_ISL_1524779	Singapore
EPI_ISL_1524778	Singapore
EPI_ISL_1367549	Singapore
EPI_ISL_1489719	Singapore
EPI_ISL_1543965	Singapore
EPI_ISL_1173251	Singapore
EPI_ISL_825082	Singapore
EPI_ISL_1897641	Singapore
EPI_ISL_1719883	Singapore

GISAID number	Location
EPI_ISL_833375	Singapore
EPI_ISL_1652101	Singapore
EPI_ISL_1098836	Singapore
EPI_ISL_857471	Singapore
EPI_ISL_1524782	Singapore
EPI_ISL_1312383	Singapore
EPI_ISL_857470	Singapore
EPI_ISL_2349770	Singapore
EPI_ISL_1098835	Singapore
EPI_ISL_1719879	Singapore
EPI_ISL_981009	Singapore
EPI_ISL_1524777	Singapore
EPI_ISL_803963	Singapore
EPI_ISL_1719878	Singapore
EPI_ISL_1519405	Singapore
EPI_ISL_1489722	Singapore
EPI_ISL_1543964	Singapore
EPI_ISL_1524781	Singapore
EPI_ISL_1719881	Singapore
EPI_ISL_1816928	Singapore
EPI_ISL_1704825	Singapore
EPI_ISL_1652102	Singapore
EPI_ISL_1315627	Singapore
EPI_ISL_995295	Singapore
EPI_ISL_1081939	Singapore
EPI_ISL_1652097	Singapore
EPI_ISL_1719880	Singapore
EPI_ISL_1519398	Singapore
EPI_ISL_1524784	Singapore
EPI_ISL_981012	Singapore
EPI_ISL_1816930	Singapore
EPI_ISL_1652098	Singapore
EPI_ISL_1312382	Singapore
EPI_ISL_2349794	Singapore
EPI_ISL_1367555	Singapore
EPI_ISL_1568432	Singapore
EPI_ISL_1620134	Singapore
EPI_ISL_1543966	Singapore
EPI_ISL_2349771	Singapore
EPI_ISL_1897640	Singapore
EPI_ISL_1367558	Singapore
EPI_ISL_1897638	Singapore
EPI_ISL_1749430	Singapore
EPI_ISL_1524783	Singapore
EPI_ISL_1543970	Singapore
EPI_ISL_1749431	Singapore
EPI_ISL_1489726	Singapore
EPI_ISL_1620140	Singapore
EPI_ISL_1620139	Singapore
EPI_ISL_1524795	Singapore
EPI_ISL_1295938	Singapore
EPI_ISL_2349832	Singapore
EPI_ISL_2508722	Singapore
EPI_ISL_2508714	Singapore
EPI_ISL_953387	Singapore
EPI_ISL_1620135	Singapore
EPI_ISL_1489723	Singapore
EPI_ISL_4115388	Japan
EPI_ISL_4115394	Japan
EPI_ISL_4115327	Japan
EPI_ISL_4115385	Japan
EPI_ISL_2335681	Japan
EPI_ISL_4115342	Japan
EPI_ISL_4115345	Japan
EPI_ISL_4115348	Japan

GISAID number	Location
EPI_ISL_10993951	Japan
EPI_ISL_10993952	Japan
EPI_ISL_10993953	Japan
EPI_ISL_10993954	Japan
EPI_ISL_10993955	Japan
EPI_ISL_10993957	Japan
EPI_ISL_825387	Japan
EPI_ISL_825391	Japan
EPI_ISL_825392	Japan
EPI_ISL_1931071	Japan
EPI_ISL_1929159	Japan
EPI_ISL_1929390	Japan
EPI_ISL_1927819	Japan
EPI_ISL_1931135	Japan
EPI_ISL_1927824	Japan
EPI_ISL_3196853	Japan
EPI_ISL_1929315	Japan
EPI_ISL_11823586	Japan
EPI_ISL_3196812	Japan
EPI_ISL_1941905	Japan
EPI_ISL_1929186	Japan
EPI_ISL_1927816	Japan
EPI_ISL_1927037	Japan
EPI_ISL_2328029	Japan
EPI_ISL_1933713	Japan
EPI_ISL_1932608	Japan
EPI_ISL_1927180	Japan
EPI_ISL_3196934	Japan
EPI_ISL_3827196	Thailand
EPI_ISL_2433378	Thailand
EPI_ISL_3827383	Thailand
EPI_ISL_2433352	Thailand
EPI_ISL_2433456	Thailand
EPI_ISL_2433401	Thailand
EPI_ISL_984304	Thailand
EPI_ISL_5030241	Thailand
EPI_ISL_2350995	Thailand
EPI_ISL_2350939	Thailand
EPI_ISL_2433387	Thailand
EPI_ISL_2433362	Thailand
EPI_ISL_4255541	Thailand
EPI_ISL_2433447	Thailand
EPI_ISL_2351073	Thailand
EPI_ISL_3826630	Thailand
EPI_ISL_2350954	Thailand
EPI_ISL_2351100	Thailand
EPI_ISL_3826563	Thailand
EPI_ISL_3827396	Thailand
EPI_ISL_2351155	Thailand
EPI_ISL_4255170	Thailand
EPI_ISL_2351062	Thailand
EPI_ISL_2351101	Thailand
EPI_ISL_2351036	Thailand
EPI_ISL_2350956	Thailand
EPI_ISL_3827237	Thailand
EPI_ISL_3827251	Thailand
EPI_ISL_3827244	Thailand
EPI_ISL_2351152	Thailand
EPI_ISL_2433548	Thailand
EPI_ISL_3826668	Thailand
EPI_ISL_2350928	Thailand
EPI_ISL_3826650	Thailand
EPI_ISL_3826648	Thailand
EPI_ISL_3826620	Thailand
EPI_ISL_3826641	Thailand

GISAID number	Location
EPI_ISL_2433461	Thailand
EPI_ISL_3892115	Thailand
EPI_ISL_2351065	Thailand
EPI_ISL_3826704	Thailand
EPI_ISL_3826720	Thailand
EPI_ISL_3827380	Thailand
EPI_ISL_3827207	Thailand
EPI_ISL_3827347	Thailand
EPI_ISL_3826524	Thailand
EPI_ISL_2678115	Thailand
EPI_ISL_3827373	Thailand
EPI_ISL_3826579	Thailand
EPI_ISL_2350960	Thailand
EPI_ISL_2351177	Thailand
EPI_ISL_877766	Thailand
EPI_ISL_3826661	Thailand
EPI_ISL_3892089	Thailand
EPI_ISL_3827254	Thailand
EPI_ISL_3826531	Thailand
EPI_ISL_2433539	Thailand
EPI_ISL_2351160	Thailand
EPI_ISL_3826664	Thailand
EPI_ISL_2433532	Thailand
EPI_ISL_2433465	Thailand
EPI_ISL_3826721	Thailand
EPI_ISL_2351047	Thailand
EPI_ISL_2433503	Thailand
EPI_ISL_3827178	Thailand
EPI_ISL_1205541	England
EPI_ISL_1205533	England
EPI_ISL_1205524	England
EPI_ISL_1205518	England
EPI_ISL_1205562	England
EPI_ISL_1205566	England
EPI_ISL_1205517	England
EPI_ISL_2739810	England
EPI_ISL_2739841	England
EPI_ISL_2740023	England
EPI_ISL_2739779	England
EPI_ISL_2739793	England
EPI_ISL_2739796	England
EPI_ISL_2753420	England
EPI_ISL_1638023	England
EPI_ISL_5566664	Philippines
EPI_ISL_5567559	Philippines
EPI_ISL_2188177	Philippines
EPI_ISL_2859726	Philippines
EPI_ISL_4741371	Philippines
EPI_ISL_2188745	Philippines
EPI_ISL_2188045	Philippines
EPI_ISL_2859714	Philippines
EPI_ISL_2859778	Philippines
EPI_ISL_5567366	Philippines
EPI_ISL_5566756	Philippines
EPI_ISL_2859720	Philippines
EPI_ISL_2155994	Philippines
EPI_ISL_2188916	Philippines
EPI_ISL_2156243	Philippines
EPI_ISL_2859269	Philippines
EPI_ISL_5567999	Philippines
EPI_ISL_4741345	Philippines
EPI_ISL_5566428	Philippines
EPI_ISL_5566378	Philippines
EPI_ISL_5568163	Philippines
EPI_ISL_5568544	Philippines

GISAID number	Location
EPI_ISL_2859679	Philippines
EPI_ISL_4712193	Philippines
EPI_ISL_5566890	Philippines
EPI_ISL_2860194	Philippines
EPI_ISL_5934900	Philippines
EPI_ISL_4712464	Philippines
EPI_ISL_5568013	Philippines
EPI_ISL_5561549	Philippines
EPI_ISL_2189162	Philippines
EPI_ISL_4459456	Philippines
EPI_ISL_4347131	United States
EPI_ISL_4347672	United States
EPI_ISL_4347113	United States
EPI_ISL_4347286	United States
EPI_ISL_4347568	United States
EPI_ISL_4347140	United States
EPI_ISL_4347237	United States
EPI_ISL_4347529	United States
EPI_ISL_4347059	United States
EPI_ISL_4347152	United States
EPI_ISL_4347070	United States
EPI_ISL_4347309	United States
EPI_ISL_4347048	United States
EPI_ISL_4347456	United States
EPI_ISL_4347366	United States
EPI_ISL_4347315	United States
EPI_ISL_4347145	United States
EPI_ISL_4347143	United States
EPI_ISL_4347259	United States
EPI_ISL_4347543	United States
EPI_ISL_4347360	United States
EPI_ISL_4347359	United States
EPI_ISL_4347696	United States
EPI_ISL_4347032	United States
EPI_ISL_4347604	United States
EPI_ISL_4347531	United States
EPI_ISL_4347495	United States
EPI_ISL_4347094	United States
EPI_ISL_4347686	United States
EPI_ISL_4347580	United States
EPI_ISL_4347448	United States
EPI_ISL_4347213	United States
EPI_ISL_4347003	United States
EPI_ISL_4347076	United States
EPI_ISL_4347093	United States
EPI_ISL_4347717	United States
EPI_ISL_4347412	United States
EPI_ISL_4347024	United States
EPI_ISL_4347169	United States
EPI_ISL_4347648	United States
EPI_ISL_4347373	United States
EPI_ISL_4347379	United States
EPI_ISL_4347633	United States
EPI_ISL_4347147	United States
EPI_ISL_4347216	United States
United States	United States
EPI_ISL_4347046	United States
EPI_ISL_4347316	United States
EPI_ISL_4347010	United States
EPI_ISL_4347036	United States
EPI_ISL_4347674	United States
EPI_ISL_4347303	United States
EPI_ISL_4347603	United States
EPI_ISL_4347385	United States
EPI_ISL_4347424	United States

GISAID number	Location
EPI_ISL_4347540	United States
EPI_ISL_4347219	United States
EPI_ISL_4347150	United States
EPI_ISL_4347535	United States
EPI_ISL_4347618	United States
EPI_ISL_4347009	United States
EPI_ISL_4347320	United States
EPI_ISL_4347698	United States
EPI_ISL_4347720	United States
EPI_ISL_4347342	United States
EPI_ISL_4347409	United States
EPI_ISL_4347054	United States
EPI_ISL_4347533	United States
EPI_ISL_4347467	United States
EPI_ISL_4347683	United States
EPI_ISL_4347042	United States
EPI_ISL_4347560	United States
EPI_ISL_4347498	United States
EPI_ISL_4347020	United States
EPI_ISL_4347158	United States
EPI_ISL_4347189	United States
EPI_ISL_4347487	United States
EPI_ISL_4347575	United States
EPI_ISL_4347361	United States
EPI_ISL_4347031	United States
EPI_ISL_4347423	United States
EPI_ISL_4347428	United States
EPI_ISL_4347116	United States
EPI_ISL_4347501	United States
EPI_ISL_4347168	United States
EPI_ISL_4347205	United States
EPI_ISL_4347545	United States
EPI_ISL_4347202	United States
EPI_ISL_4347253	United States
EPI_ISL_4347630	United States
EPI_ISL_4347561	United States
EPI_ISL_4347386	United States
EPI_ISL_4347453	United States
EPI_ISL_4347635	United States
EPI_ISL_4347333	United States
EPI_ISL_4347466	United States
EPI_ISL_4347268	United States
EPI_ISL_4347124	United States
EPI_ISL_4347589	United States
EPI_ISL_4347159	United States
EPI_ISL_4347430	United States
EPI_ISL_4347214	United States
EPI_ISL_4347395	United States
EPI_ISL_4347459	United States
EPI_ISL_4347328	United States

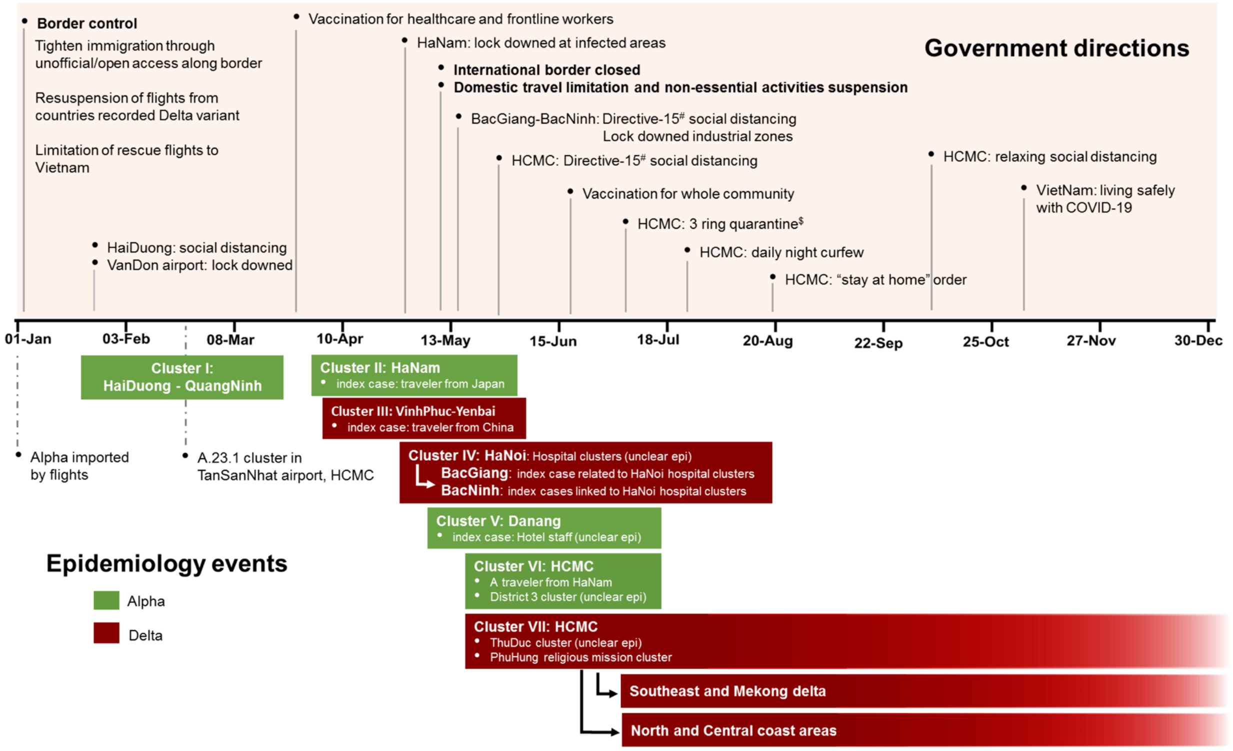

Appendix Figure 1. Government directions and COVID-19 epidemiology events in Vietnam in 2021. #Directive-15: Suspension of non-essential services/businesses and mass gatherings, applying physical distance of 2 m when contact with others, banning the gatherings of 20 persons or more in one place and 10 persons or more outside workplaces and limitation of movements. $3 ring quarantine: apply Directive-16 whole city (bans gatherings of two more persons in public and asks persons to only leave homes for emergencies, food, medicine, work in factories, and businesses that involve essential goods and services), lockdown at residential areas with covid-19 case report, home quarantine for F1. HCMC: Ho Chi Minh city.

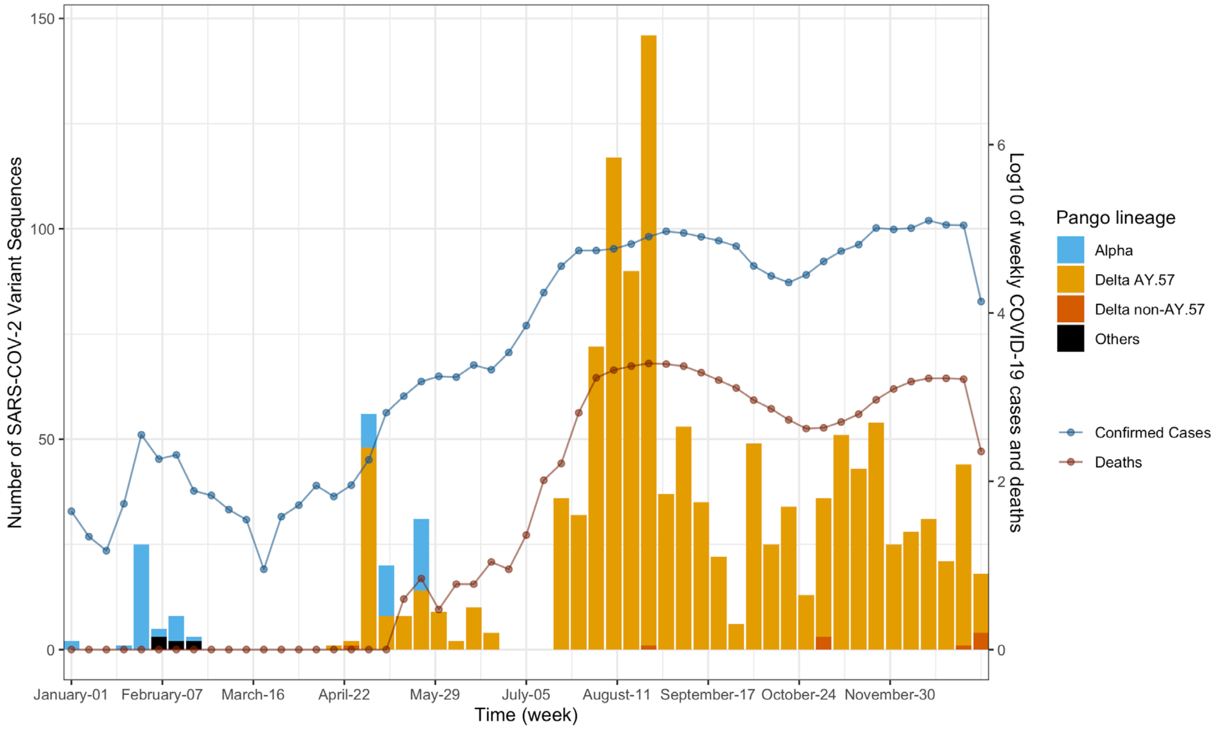

Appendix Figure 2. Line chart showing the number of reported COVID-19 cases and deaths during the 2021 outbreak of COVID-10 in Vietnam alongside the monthly number of SARS-CoV-2 variants (bar chart) detected among cases of community transmission between January and December 2021. Delta variant non-AY.57 lineage includes B.1.617.2 (n = 1), AY.23 (n = 3), AY.79 (n = 3), AY.85 (n = 1), AY.6 (n = 1), and AY.38 (n = 1). Others include lineages B.1.637 (n = 2) and A.23.1 (n = 5).

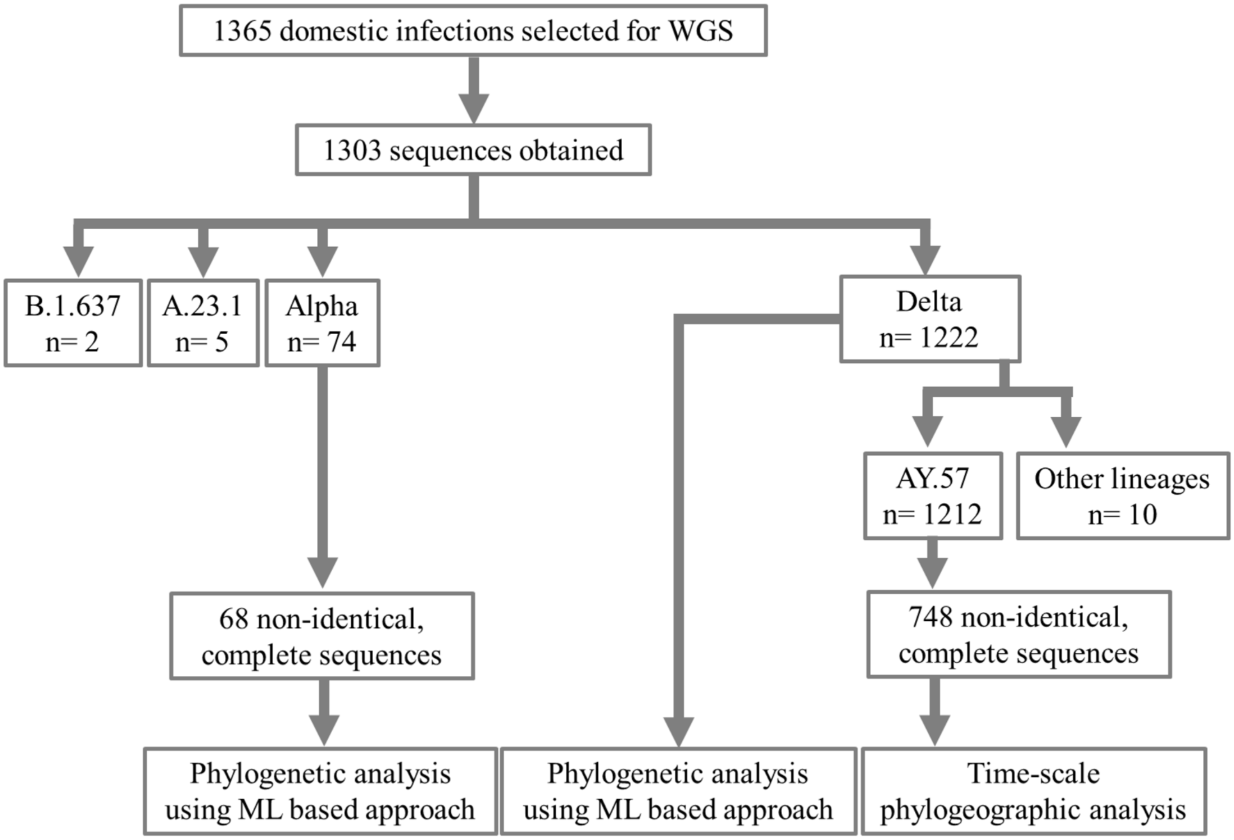

Appendix Figure 3. Workflow of the study.

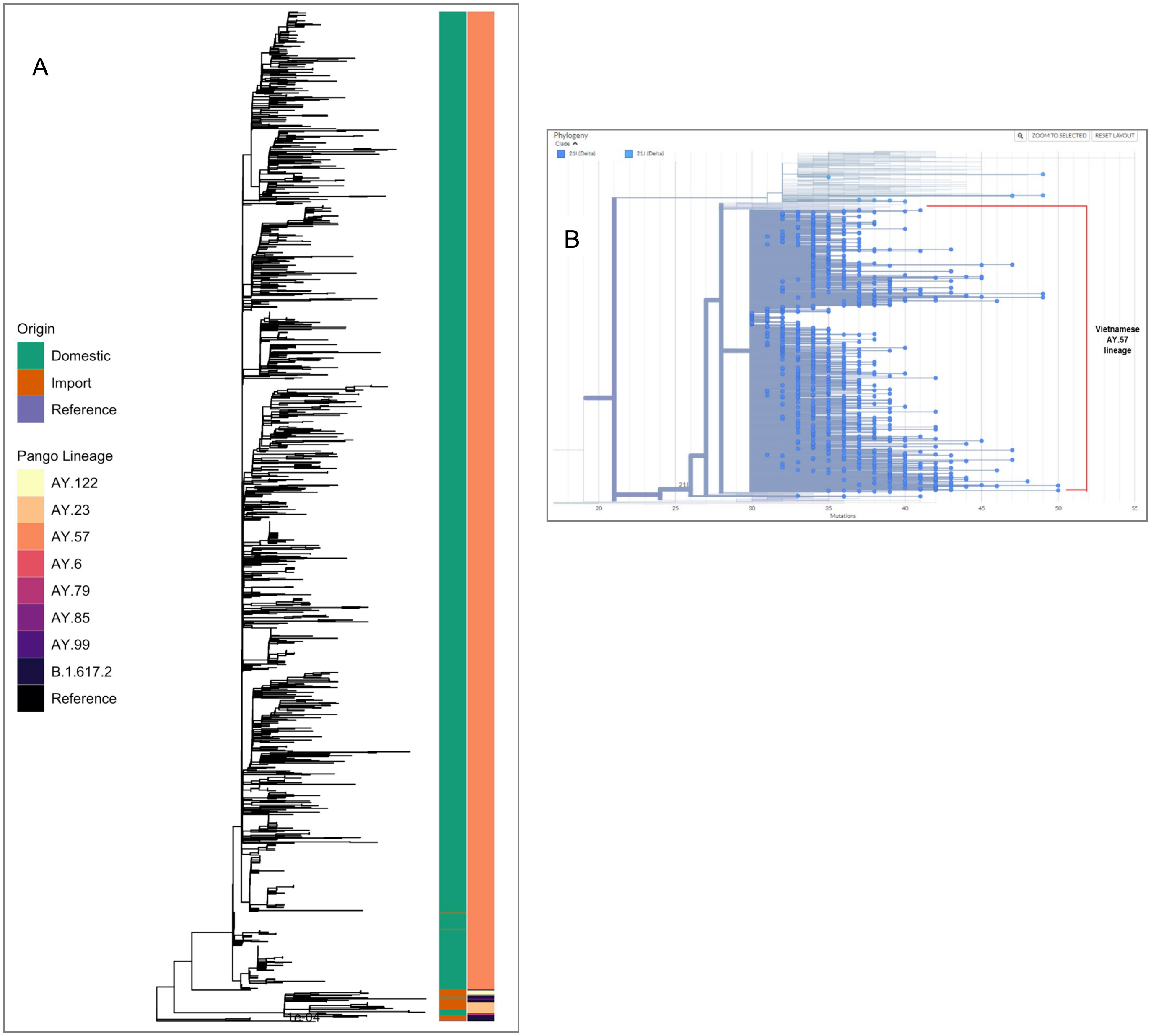

Appendix Figure 4. A) Reconstructed ML tree depicting the relationship between Delta variants detected in Vietnam, and **B)** NexClade Based phylogenetic analysis illustrating the placement of the Vietnamese sequences among global sequences submitted GISAID.

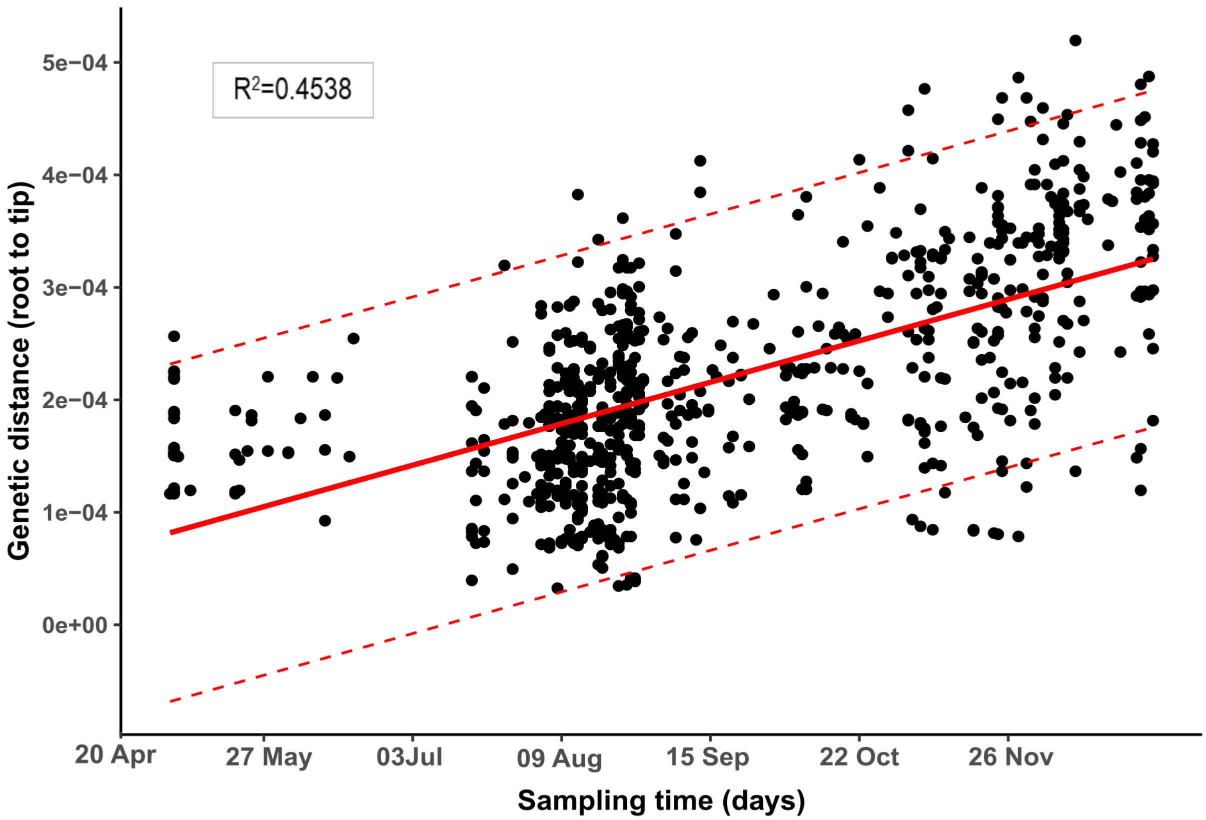

Appendix Figure 5. Root to tip regression of AY.57 coding sequences using for the evolution analysis. The solid line indicates the regression line; the dotted lines represent upper and lower limits of 95% confidence interval. Outliers were excluded from subsequence spatiotemporal evolutionary analysis. TempEst analysis indicated a positive correlation between genetic divergence and sampling time; the retrieved R^2 value of 0.4538 (F = 438.4117, p value <0.001), suggesting a moderate temporal signal in the included sequences (https://doi.org/10.1093/molbev/msr121).
